# Challenges in monitoring the quality of care in multiple sclerosis—authors' reply

**DOI:** 10.1016/j.lanepe.2024.100951

**Published:** 2024-05-27

**Authors:** Isabel Voigt, Stefanie Fischer, Undine Proschmann, Urszula Konofalska, Peggy Richter, Hannes Schlieter, Thomas Berger, Sven G. Meuth, Hans-Peter Hartung, Katja Akgün, Tjalf Ziemssen

**Affiliations:** a Center of Clinical Neuroscience, Department of Neurology, Medical Faculty and University Hospital Carl Gustav Carus, TUD Dresden University of Technology, Fetscherstraße 74, 01307, Dresden, Germany; b Research Group Digital Health, Faculty of Business and Economics, TUD Dresden University of Technology, 01062, Dresden, Germany; c Department of Neurology, Medical University of Vienna, Währinger Gürtel 18-20, 1090, Vienna, Austria; d Comprehensive Center for Clinical Neurosciences & Mental Health, Medical University of Vienna, Währinger Gürtel 18-20, 1090, Vienna, Austria; e Department of Neurology, Medical Faculty and University Hospital Düsseldorf, Heinrich-Heine-University Düsseldorf, Moorenstr. 5, 40225, Düsseldorf, Germany

We appreciate Zettl et al.‘s^1^ insightful observations and reflections in their correspondence to our paper “Consensus quality indicators for monitoring multiple sclerosis”.^2^ We value any concerns and critical evaluations that enrich the discussion on quality indicators (QIs) for improving care for people with MS (pwMS). With the following response we want to state our position on the individual points of criticism.

Zettl et al. criticize that the developed QIs do not cover diagnostic confirmation. While acknowledging the significance of this concern and the challenge of misdiagnosis in MS, our focus was specifically on QIs related exclusively to the monitoring of MS which seemed to be the best first step in our pioneering activity of QI implementation in MS.^3^ This approach assumes a confirmed diagnosis as a starting point, and therefore, diagnosis was not a component of our examination. Designing QIs for the complex diagnostic process is inherently challenging due to the variability in diagnostic steps and the multitude of possible alternative diagnoses. Nonetheless, we recognize the need to improve quality management in diagnosis, which our QI team will address in later stages. We believe that our efforts in developing digital pathways for MS management represent a step in the right direction towards addressing this issue.^4^

Zettl et al.‘s comments regarding the limitations of the EDSS as a disability measurement tool highlight important aspects of disability assessment in MS. Addressing these limitations and exploring more comprehensive measures could optimize disease monitoring and patient care.^5^ However, we firmly believe that it is preferable to utilize a standard tool, despite its limitations, than to forgo the use of any standardized tool due to a lack of better alternatives. This is especially crucial because quality measurement inherently relies on structured and preferably standardized data. In pursuit of this goal, we are dedicated to developing a digital twin platform that integrates data to optimize disability measurement.^6^ This platform will leverage advanced technology to enhance the accuracy and effectiveness of disability assessment in MS care, aligning with the imperative for structured and standardized quality measurement.

Furthermore, Zettl et al. criticize the lack of explanation regarding querying medications and the underrepresentation of polypharmacy. We clarify that these aspects are addressed in our QIs. Specifically, we have developed a dedicated QI focused on inquiring about comorbidities and medications. This QI involves the initial steps of querying all comorbidities and medications, encompassing disease-modifying therapies (DMT), symptomatic therapies, adjuvants, and complementary or alternative therapies. The subsequent reassessment of therapy goals, which constitutes the third step of our QI, is a critical component. This reassessment may lead to adjustments in therapy goals based on the comprehensive evaluation of patient needs and treatment outcomes. Importantly, this process includes consideration of undesirable side effects, potential drug interactions, and other key factors. The reassessment within our QI framework is designed to ensure the safety, effectiveness, and appropriateness of therapeutic interventions, considering the complex interplay of medications and patient-specific factors. However, monitoring drug interventions was not specifically part of our work. We view monitoring drug therapies as a separate process, similar to diagnosis. While diagnosis, monitoring, and therapy are interconnected in clinical practice, our approach for developing QIs focused on delineating these processes for clarity and effectiveness in quality measurement. Moving forward, additional efforts are needed to develop specific digital pathways for monitoring drug therapies.

We appreciate Zettl et al.'s suggestion to include patient centered QIs, such as waiting times and overall satisfaction. Integrating the patient's perspective is crucial for us, because in many instances, we can ultimately let the patient control the processes through these QIs. In specialized centers, we observe a trend towards continuous improvement, but in facilities with lower quality MS care, data and improvement efforts are lacking. We consider patient-side QIs to be crucial for enhancing care quality. This can only be achieved if patients have the ability to work with QIs and integrate them into their healthcare management. Patient-centered metrics are indeed essential to capture the full spectrum of quality care, and we have already considered this aspect. As outlined in our work's impact section, we plan to involve the patient's perspective in our pilot study and to present this on the MS patient portal.^7^ We recognize the importance of incorporating patient feedback and experiences to enhance the quality measurement process and ensure that care is aligned with patient needs and preferences. We appreciate the emphasis of patient-centered metrics, and look forward to incorporating these perspectives into our ongoing QI improvement efforts.

Overall, we appreciate the valuable insights and constructive feedback, which will guide our efforts to improve the quality of care for pwMS. We thank the authors for their contributions to advancing this important field of healthcare.

## Contributors

IV: conceptualization, writing–original draft, writing–review & editing. SF, UP, UK, PR, HS, TB, SGM, HPH: writing–review & editing. TZ: conceptualization, supervision, writing–review & editing.

## Declaration of interests

IV, SF, UK, HS, PR and HPH declare that the research was conducted in the absence of any commercial or financial relationships that could be construed as a potential conflict of interest.

UP received personal consulting fees service from Biogen, Roche and Sanofi and personal payment for Speakers bureaus from Novartis, Merck, Biogen, Bayer and Roche. TB received unrestriceted grants to his institution from Biogen, Bristol-Myers-Squibb, Merck, Novartis, Roche, Sanofi/Genzyme, and TEVA ratiopharm; payments for participation in clinical trials made to his Institution from Alexion, Bayer, Biogen, Bristol-Myers-Squibb, Merck, Novartis, Octapharma, Roche, Sanofi/Genzyme, and TEVA; personal consulting fees from Almirall, Bionorica, Horizon, Merck, Novartis, Roche, Sandoz, Sanofi; personal payment for lectures, presentations, speakers bureaus, manuscript writing and educational events from Almirall, Bayer, Biogen, Biologix, Bionorica, Bristol-Myers-Squibb, Eisai, GW Pharma, Horizon, Janssen-Cilag, MedDay, Merck, Novartis, Octapharma, Roche, Sandoz, Sanofi/Genzyme, TG Pharmaceuticals, TEVA-ratiopharm and UCB. SGM receives honoraria for lecturing, and travel expenses for attending meetings from Academy 2, Argenx, Alexion, Almirall, Amicus Therapeutics Germany, Bayer Health Care, Biogen, BioNtech, BMS, Celgene, Datamed, Demecan, Desitin, Diamed, Diaplan, DIU Dresden, DPmed, Gen Medicine and Healthcare products, Genzyme, Hexal AG, Impulze GmbH, Janssen Cilag, KW Medipoint, MedDay Pharmaceuticals, Merck Serono, MICE, Mylan, Neuraxpharm, Neuropoint, Novartis, Novo Nordisk, ONO Pharma, Oxford PharmaGenesis, Roche, Sanofi-Aventis, Springer Medizin Verlag, STADA, Chugai Pharma, QuintilesIMS,Teva, Wings for Life international and Xcenda. His research is funded by the German Ministry for Education and Research (BMBF), Bundesinstitut für Risikobewertung (BfR), Deutsche Forschungsgemeinschaft (DFG), Else Kröner Fresenius Foundation, Gemeinsamer Bundesausschuss (G-BA), German Academic Exchange Service, Hertie Foundation, Interdisciplinary Center for Clinical Studies (IZKF) Muenster, German Foundation Neurology and Alexion, Almirall, Amicus Therapeutics Germany, Biogen, Diamed, DGM e.v., Fresenius Medical Care, Genzyme, Gesellschaft von Freunden und Förderern der Heinrich-Heine-Universität Düsseldorf e.V., HERZ Burgdorf, Merck Serono, Novartis, ONO Pharma, Roche, and Teva.

KA received personal compensation from Roche, Sanofi, Novartis, Merck, Teva, BMS for consulting or speaker service.

TZ received personal research support from Biogen, Novartis, Merck, Sanofi; personal consulting fees from Biogen, Roche, Novartis, Celgene and Merck and personal payment for speakers bureaus from Roche, Novartis, Merck, Sanofi, Celgene, and Biogen.
